# New twists in the unfolded protein response

**DOI:** 10.7554/eLife.00243

**Published:** 2012-10-15

**Authors:** Benedict C S Cross, David Ron

**Affiliations:** **Benedict C S Cross** is at the University of Cambridge Metabolic Research Laboratories and the NIHR Cambridge Biomedical Research Centre, Cambridge, United Kingdombcc33@medschl.cam.ac.uk; **David Ron** is an *eLife* reviewing editor, and is at the University of Cambridge Metabolic Research Laboratories and the NIHR Cambridge Biomedical Research Centre, Cambridge, United Kingdomdr360@medschl.cam.ac.uk

**Keywords:** Unfolded Protein Response, Ire1, selective mRNA decay, Bip1 mRNA stabilization, ER homeostasis

## Abstract

The response of *S. pombe*, also known as fission yeast, to misfolded proteins involves mechanisms that have not been observed in other species.

**Related research article** Kimmig P, Diaz M, Zheng J, Williams C, Lang A, Aragón T, Li H, Walter P. 2012. The unfolded protein response in fission yeast modulates stability of select mRNAs to maintain protein homeostasis. *eLife* **1**:e00048. doi: 10.7554/eLife.00048

**Image** Cleavage of mRNAs by the protein-folding sensor IRE1

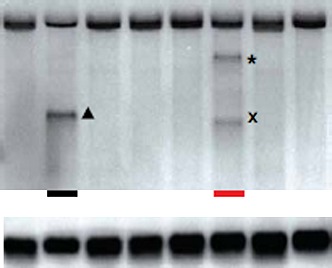

In eukaryotic cells many proteins are synthesised in the endoplasmic reticulum (ER), and within each cell this production process must be carefully synchronised with secretion and the export of proteins from the ER. Any failure to maintain a balance between the production of proteins—which involves strings of amino acids folding into specific shapes—and their departure from the ER will lead to errors in the synthesis process and, eventually, to toxicity through a phenomenon known as ‘ER stress’. The collection of mechanisms that cells use to combat ER stress is known as the unfolded protein response. Writing in *eLife*, Philipp Kimmig, Marcy Diaz and co-workers at the University of California at San Francisco (UCSF) report the results of experiments on *Schizosaccharomyces pombe*, a fission yeast, that reveal aspects of the unfolded protein response that have not been observed before (Kimmig et al., 2012).

In all systems studied to date, cells respond to an accumulation of misfolded proteins in the ER by producing more protein-folding machinery within the ER. This process is initiated by a stress transducer—either IRE1, PERK or ATF6—that spans the membrane between the ER and the rest of the cell (Walter and Ron, 2011). IRE1, which is conserved in all eukaryotes, is a dual-function enzyme that detects unfolded proteins in the ER and then sends signals to the nucleus of the cell to address this problem.

In metazoans, plants and budding yeast, this signalling involves the IRE1 removing a short fragment from within a strand of messenger RNA (mRNA)—a process known as mRNA splicing. This short fragment is degraded and the two flanking pieces of mRNA are joined together to encode an active transcription factor that specifically increases the expression of the proteins that make up the protein-folding machinery found in the ER. Until recently it was generally agreed that the primary function of IRE1 during ER stress involved this transcriptional activation, and it was also thought that this process required a specific mRNA substrate (XBP1 for metazoans, bZIP60 for plants, and HAC1 for budding yeast). Fission yeast was known to be an exception to this appealing unity because no analogous IRE1 substrate had ever been identified (Figure 1A).

**Figure 1.**
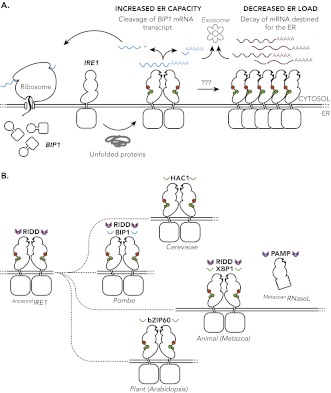
The unfolded protein response in *S. pombe* and other species. (**A**) The accumulation unfolded proteins in the endoplasmic reticulum (ER) of *S. pombe* leads to activation of IRE1 (presumably by nucleotide binding (green), auto-phosphorylation (red) and the formation of dimers), which is turn leads to the cleavage of mRNAs in the cytosol. The subsequent degradation of the cleaved mRNAs (known as RIDD) and explusion from the cell (via the exosome) reduced the protein-folding load on the ER. However, as described in the text, the mRNA that encodes for the molecular chaperone *Bip1* escapes this fate: although it is cleaved at the 3′ untranslated region (shown by the asterisk), the 5′ *Bip1* mRNA fragment is first stabilized and then translated into *Bip1* by the ribosome. The enhanced production of *Bip1* helps to increase the protein-folding capacity of the ER. The same IRE1 dimer can perform both *Bip1* mRNA cleavage and RIDD: however, larger IRE1 oligomers (right) can also perform RIDD in a second level of the unfolded protein response. (**B**) Ancestral IRE1 may have possessed RIDD activity prior to the evolution of the yeasts (top), animals (middle) and plants (bottom). This vestigial RIDD function is not evident in budding yeast (*S. cerevisiae*), and it is not known if it exists in plants (*Arabidopsis*), but it is evident in the animals (metazoans). However, in all three cases IRE1 exerts transcriptional control by splicing mRNA to regulate the expression of various transcription factors (HAC1, XBP1 and bZIP60). The unfolded protein response in fusion yeast (*S. Pombe*) is different in that it involves RIDD and the direct post-transcriptional stabilisation of the molecular chaperone *Bip1*. RNase L is a distant relative of IRE1 that triggers mRNA decay in mammals in a way that is not dissimilar to RIDD.

In 2006 it was discovered that ER stress in drosophila cells leads to the decay of a number of ER-localised mRNAs (Hollien and Weissman, 2006), and since then it has become apparent that cleavage of the mRNAs by IRE1 is the first step, and also the limiting step, in their degradation. This process, later termed regulated IRE1-dependent decay, has also been found in mammalian cells and is thought to reduce the protein-folding load by prompting the pre-emptive degradation of mRNAs that are destined for the ER (Hollien et al., 2009). This finding, which came as a surprise at the time, set the scene for the latest work on the unfolded protein response in fission yeast.

Kimmig and co-workers, working in Peter Walter’s lab at UCSF, conducted a transcript analysis of ER-stressed *S. pombe* cells and found a distinct decrease in the abundance of mRNAs encoding for proteins that were destined for the ER (Kimmig et al., 2012). Further molecular analysis confirmed IRE1-dependent cleavage of this subset of mRNAs, followed by degradation of the 5′ and 3′ fragments: these are the hallmarks of regulated IRE1-dependent decay (RIDD), and they suggest that this process might provide the foundation of the unfolded protein response in these cells.

The sites of the IRE1-dependent mRNA cleavage in *S. pombe* share some sequence homology with those of other species, but not enough to account entirely for the apparent specificity of RIDD. In fact, the difficulty in identifying clear rules for RIDD in this study and others had raised a number of questions about the phenomenon itself. Is RIDD a *bona fide* functional output of the unfolded protein response? Or is it an irrelevant but inevitable corollary of a potent ER-embedded RNase? Whilst budding yeast appear to survive without RIDD, the UCSF team makes a strong genetic case for its importance in fission yeast, estimating that RIDD accounts for a 15% decrease in unfolded protein load on the ER of wildtype fission yeast. Although this is a modest decrease, it is worth noting that the PERK branch of the unfolded protein response in the active secretory cells of the mammalian pancreas makes an important contribution to cell survival, even though it only reduces the unfolded protein load by an estimated 30% (Harding et al., 2001). In contrast, cultured mammalian cells can survive acute ER stress without the help of the IRE1 transcriptional branch (Cross et al., 2012).

These new findings in *S. pombe* also highlight the potential for selective RIDD to lead to a qualitative change in cell physiology, in addition to a quantitative reduction in the unfolded protein load. The selective decay of transcripts that are involved in sterol metabolism is a salient example of this, since sterol accumulation promotes ER stress in mammalian cells (Feng et al., 2003) and budding yeast (Pineau et al., 2009).

The behaviour of a protein called *Bip1* provided an unexpected twist in the story of the unfolded protein response of *S. pombe*. *Bip1* is a molecular chaperone, a protein that helps other proteins or macromolecules to fold and unfold in cells, and Kimmig, Diaz and co-workers found that the mRNA that encodes for *Bip1* was elevated in wildtype ER-stressed *S. pombe*. Most species increase the expression of molecular chaperones by increasing the rate of transcription of their encoding mRNA. The researchers found that the response in *S. pombe* was post-transcriptional. In particular they found that the *Bip1* mRNA was cleaved at a typical RIDD site in the 3′ untranslated region (indicated by an asterisk in Figure 1A). However, unlike all other known RIDD targets, which are rapidly degraded following cleavage, the 5′ mRNA fragment that contains the coding sequence for the *Bip1* molecular chaperone is stabilised, which leads to the increased production of *Bip1* and, therefore, to increased protein-folding capacity in the ER.

The catalytic residues in IRE1 are well conserved between *S. Pombe* and other species, and the UCSF results suggest that IRE1 is directly responsible for the cleavage of *Bip1* mRNA in *S. pombe*. Whilst much is yet to be understood about the mechanism underlying the counter-intuitive stabilization of the 5′ fragment, the output is clear and represents a novel post-transcriptional approach to combating ER stress.

These findings also illuminate the evolution of the unfolded protein response (Figure 1B). The common shared biology appears to be the cleavage and splicing of RNA by IRE1, with the biomolecular apparatus that couples this activity to the regulated expression of a transcription factor emerging only later. In plants, for example, this splicing deletes a region of mRNA that codes for a specific transmembrane structure, which liberates a tethered transcription factor from the ER (Deng et al., 2011). In metazoans and budding yeast the activity of IRE1 is to control the translation of an active transcription factor. And the appearance of RNase L, a distant relative of IRE1 that is involved in innate immunity in mammals (Malathi et al., 2007), appears to be a late reversion to the sort of behaviour displayed by ancestral IRE1. Indeed, given the mechanistic diversity for dealing with unfolded proteins in the ER that is highlighted by the UCSF work, the similarities in the responses of budding yeast (*S. cerevisiae*) and the metazoans is quite remarkable. It will be fascinating to discover how the unfolded protein response has evolved in each lineage.
